# Caspofungin Induced Cell Wall Changes of *Candida* Species Influences Macrophage Interactions

**DOI:** 10.3389/fcimb.2020.00164

**Published:** 2020-05-12

**Authors:** Louise A. Walker, Carol A. Munro

**Affiliations:** School of Medicine, Medical Sciences and Nutrition, Institute of Medical Sciences, University of Aberdeen, Aberdeen, United Kingdom

**Keywords:** chitin, β-1, 3-glucan, GPI-anchored proteins, fungal cell wall, echinocandin, macrophages, immune response

## Abstract

*Candida* species are known to differ in their ability to cause infection and have been shown to display varied susceptibilities to antifungal drugs. Treatment with the echinocandin, caspofungin, leads to compensatory alterations in the fungal cell wall. This study was performed to compare the structure and composition of the cell walls of different *Candida* species alone and in response to caspofungin treatment, and to evaluate how changes at the fungal cell surface affects interactions with macrophages. We demonstrated that the length of the outer fibrillar layer varied between *Candida* species and that, in most cases, reduced fibril length correlated with increased exposure of β-1,3-glucan on the cell surface. *Candida glabrata* and *Candida guilliermondii*, which had naturally more β-1,3-glucan exposed on the cell surface, were phagocytosed significantly more efficiently by J774 macrophages. Treatment with caspofungin resulted in increased exposure of chitin and β-1,3-glucan on the surface of the majority of *Candida* species isolates that were tested, with the exception of *C. glabrata* and *Candida parapsilosis* isolates. This increase in exposure of the inner cell wall polysaccharides, in most cases, correlated with reduced uptake by macrophages and in turn, a decrease in production of TNFα. Here we show that differences in the exposure of cell wall carbohydrates and variations in the repertoire of covalently attached surface proteins of different *Candida* species contributes to their recognition by immune cells.

## Introduction

*Candida* species differ in their ability to cause infection. *Candida albicans* is the most common cause of *Candida* bloodstream infections (40%), followed by *Candida glabrata* (29%), *Candida parapsilosis* (11%), *Candida tropicalis* (4%), *Candida dubliniensis* (2%), and *Candida lusitaniae* (<1%) (Data captured from England; Health Protection Report, 2018). *Candida* species also have varied susceptibilities to antifungal drugs. The echinocandins act by specifically inhibiting the synthesis of β-1,3-glucan in the fungal cell wall. The inhibition of β-1,3-glucan synthesis occurs predominantly through inhibition of the catalytic Fks glucan synthase subunits (Kurtz and Douglas, 1997). Caspofungin is one of the most widely used of the echinocandins in the clinic and has fungicidal activity against the majority of *Candida* species. *C. lusitaniae, C. parapsilosis*, and *Candida guilliermondii* are known to have relatively reduced susceptibility compared to *C. albicans* and in recent years the incidence of clinical isolates of *C. glabrata*, which have acquired resistance to the echinocandins has increased (Garcia-Effron et al., 2010; Pfaller et al., 2013). Alarmingly echinocandin-resistant *C. glabrata* isolates (up to 38%) were also cross-resistant to fluconazole (Pfaller et al., 2012, 2013). Acquired resistance is predominantly mediated by point mutations within hotspot regions in the *FKS* genes (Park et al., 2005; Balashov et al., 2006; Garcia-Effron et al., 2010; Alexander et al., 2013; Pham et al., 2014; Marti-Carrizosa et al., 2015).

The fungal cell wall determines cell shape, maintains cell wall integrity and is recognized by the innate immune system. The cell walls of *Candida* spp. in general are composed of an inner core of chitin and β-1,3-glucan, which is covered by an outer layer of cell wall proteins, the majority of which are covalently linked to β-1,6-glucan by modified glycosylphosphatidylinositol (GPI) anchors (Gow et al., 2017). The cell wall is a dynamic structure which alters its composition in response to cell wall stress by upregulating genes involved in cell wall synthesis, in an attempt to restore the robustness of the cell wall (Walker et al., 2008). Treatment of *C. albicans* with caspofungin has been shown to lead to a compensatory increase in chitin content, *in vitro* and *in vivo* (Walker et al., 2008; Lee et al., 2012). This compensatory increase in chitin is not specific to *C. albicans* as *C. tropicalis, C. parapsilosis, C. guilliermondii*, and isolates of *C. krusei* also demonstrated an elevation in chitin content in response to caspofungin treatment (Walker et al., 2013). In addition, isolates of *C. albicans, C. krusei, C. parapsilosis*, and *C. guilliermondii*, which have increased chitin content are less susceptible to caspofungin (Walker et al., 2008, 2013). *C. albicans* cells with elevated chitin contents have also been shown to be less susceptible to caspofungin in a murine model of systemic infection (Lee et al., 2012).

Putative GPI-modified cell wall proteins have been implicated in susceptibility to caspofungin as deletion of specific proteins leads to alterations in cell wall composition and subsequently to differences in susceptibility to caspofungin (Plaine et al., 2008). As a result of the cell wall remodeling that occurs in response to caspofungin treatment, chitin and β-1,3-glucan also become more exposed on the cell surface (Wheeler and Fink, 2006; Wheeler et al., 2008; Mora-Montes et al., 2011).

The fungal cell wall plays an important role in immune recognition as it is the first point of contact between the host and pathogen. The main innate immune cells that are involved in the recognition of invading pathogens are neutrophils, monocytes and macrophages (Netea et al., 2008). Components of the cell wall act as pathogen associated molecular patterns (PAMPs), which are recognized by pattern recognition receptors (PRRs) on host cells (Brown and Gordon, 2001; Porcaro et al., 2003; Kohatsu et al., 2006; McGreal et al., 2006; Netea et al., 2006, 2008). The two main classes of PRRs are the Toll-like receptors (TLRs) and the C-type lectin receptors (CLRs). The TLRs recognize phospholipomannan and *O*-linked mannan, whereas the C-type lectin receptors recognize β-1,3-glucan and mannan (Stahl et al., 1978; Wileman et al., 1986; Jouault et al., 2003; McGreal et al., 2006; Netea et al., 2006, 2008; Sato et al., 2006; Gow et al., 2007; Taylor et al., 2007). The increased exposure of chitin and β-1,3-glucan on the cell surface of *C. albicans*, in response to caspofungin treatment, results in altered cytokine production by immune cells, indicating that remodeling of the cell wall influences interactions with host cells (Wheeler and Fink, 2006; Wheeler et al., 2008; Mora-Montes et al., 2011; Baltch et al., 2012a,b; Fidan et al., 2014). Caspofungin treatment of *C. albicans* cells, followed by UV inactivation led to increased recognition of fungal cells by the C-type lectin, Dectin-1, which in turn increased cytokine production (Wheeler and Fink, 2006; Wheeler et al., 2008). In contrast, increased exposure of chitin on the surface of *C. albicans* has been shown to result in reduced cytokine production (Mora-Montes et al., 2011). Naturally occurring variations in the cell walls of different *C. albicans* isolates influences the dependency on dectin-1 for recognition and clearance of fungal cells (Marakalala et al., 2013). In addition the role of dectin-1 in recognition of four different *Candida* species, with differences in their cell wall carbohydrate composition, also varies (Thompson et al., 2019).

Because different *Candida* species vary in their ability to cause infection and vary in their cell wall composition, we aimed to perform a detailed comparison of the cell wall architectures of seven most prevalent *Candida* spp. and examine how their surface carbohydrates and proteomes change in response to caspofungin (CSF) treatment. In addition, the effect of caspofungin-induced cell wall changes of the different *Candida* species on the response of host immune cells was investigated.

## Materials and Methods

### Strains, Media, and Growth Conditions

The strains of each *Candida* species that were used in this study had been typed and their genomes sequenced, these strains are listed in Table 1. Strains were maintained on solid YPD medium [1% (w/v) yeast extract, 2% (w/v) mycological peptone, 2% (w/v) glucose, 2% (w/v) agar]. RPMI-1640 medium (Gibco, Paisley, UK) was used for drug susceptibility testing and for growing cultures for HPLC and proteomic analysis.

**Table 1:** Caspofungin susceptibilities of sequenced strains of different *Candida* species^a^.

**Species**	**Strain name**	**Caspofungin IC_**50**_ (μg/ml)**	**References**
*Candida albicans*	SC5314	0.032	(Gillum et al., 1984)
*Candida dubliniensis*	CD36	0.064	(Sullivan et al., 1995)
*Candida glabrata*	ATCC2001	0.064	American Type Culture Collection
*Candida tropicalis*	MYA-3404	0.125	American Type Culture Collection
*Candida lusitaniae*	ATCC42720	0.5	American Type Culture Collection
*Candida parapsilosis*	ATCC22019	1	American Type Culture Collection
*Candida guilliermondii*	ATCC6260	8	American Type Culture Collection

a *As determined by broth microdilution testing. The IC_50_ was defined as the concentration of caspofungin that inhibited 50% growth of each Candida species when cultured in RPMI-1640 for 24 h at 37°C*.

### Preparation of FITC-Stained *Candida* spp

Staining of *Candida* spp. yeast cells with fluorescein isothiocyanate (FITC) was performed as previously described (Graham et al., 2006). Briefly, *Candida* spp. were grown in YPD in the presence or absence of an IC_50_ concentration of caspofungin (Walker et al., 2013) in YPD at 30°C for 6 h. *Candida* spp. cells were washed twice with PBS and stained for 10 min at room temperature in the dark with 1 mg/ml fluorescein isothiocyanate (FITC) (Sigma, Dorset, United Kingdom) in 0.05 M carbonate-bicarbonate buffer (pH 9.6) (BDH Chemicals, VWR International, Leicestershire, United Kingdom). Cells were then washed three times in 1x PBS to remove residual FITC and were resuspended in 1x PBS. FITC-stained 6x10^5^ *Candida* spp. cells that had been pre-grown with or without IC_50_ caspofungin were then added to the macrophages at a multiplicity of infection (MOI) of 3 *Candida* spp. cells per macrophage, and incubated at 37°C for 4 h before visualization.

### Phagocytosis and Cytokine Assays

#### Macrophage Cell Culture

J774.1 murine macrophages (European Collection of Cell Culture) were cultured in Dulbecco's modified Eagle's medium (DMEM; Lonza Group, Ltd., Braine-l'Alleud, Belgium), supplemented with 10% (vol/vol) fetal calf serum, 2% (wt/vol) penicillin and streptomycin antibiotics (Invitrogen, Ltd., Paisley, United Kingdom) in tissue culture flasks (Nalge Nunc, International, Hereford, United Kingdom) at 37°C and 5% (vol/vol) CO_2_. For phagocytosis assays, 2 × 10^5^ J774.1 macrophages in 2 ml supplemented DMEM medium were seeded onto glass-based imaging dishes [Imaging dish CG 1.0; MACS Miltenyi Biotec (130-098-282), Surrey, UK] and incubated at 37°C with 5% CO_2_ for at least 2 h to allow for macrophage adherence to the dish. Immediately prior to experiments, DMEM medium was replaced with 2 ml pre-warmed supplemented CO_2_-independent medium (Gibco, Invitrogen, Paisley, UK) and cultures used immediately for phagocytosis assays.

After 4 h of co-incubation 50 μl of supernatant was removed from phagocytosis assays to allow determination of TNFα production from J774 macrophages as a result of incubation with each *Candida* species. TNFα concentrations were determined using enzyme-linked immunosorbent assays (R&D Systems) according to the manufacturer's instructions.

### Cell Wall Staining of Exposed Chitin and Glucan

*Candida* spp. yeast cells were grown in YPD in the presence or absence of an IC_50_ concentration of caspofungin in YPD at 30°C for 6 h. Cells were stained with 5 μg/ml Fc:Dectin1 protein to visualize exposed β-1,3-glucan, as previously described (52). Briefly, 1 × 10^6^ cells/sample were blocked with FACS block (0.5% BSA, 5% HI-rabbit serum, 5 mM ETDA, 2 mM NaAzide in PBS) for 30 min. Cell pellets were harvested and washed 3 × with 1 ml FACS wash (0.5% BSA, 5 mM EDTA, 2 mM NaAzide in PBS) at 4°C. After washing, the cell pellet was resuspended in 100 μl of 5 μg/ml Fc:Dectin1 protein (diluted from stock in FACS block) and incubated for 1 h on ice. Cells were then washed 3 times with 1 ml FACS wash and resuspended in 200 μl FACS block plus 1/200 anti-human Fc+Alexa-488 and incubated for 45 min on ice. After incubation cells were washed three times with 1 ml FACS wash and fixed with 200 μl 1% formaldehyde. After fixing cells were also stained with 25 μg/ml Wheat Germ Agglutinin conjugated to Texas Red (WGA-TR). All samples were examined by DIC and fluorescence microscopy using a Zeiss Axioplan 2 microscope. Images were recorded digitally using the Openlab system (Openlab v 4.04, Improvision, Coventry, UK) and a Hamamatsu C4742- 95 digital camera (Hamamatsu Photonics, Hamamatsu, Hertfordshire, UK). In all experiments the exposure time for a series of fluorescence images was fixed so the intensity of fluorescence relative to a control was used as standard. Chitin and β-1,3-glucan exposure were measured by quantifying WGA-TR and Fc:Dectin1-488 fluorescence, respectively, of individual yeast cells (Walker et al., 2008). Mean fluorescence intensities were calculated for 50 individual cells of each *Candida* species, for each condition.

### High-Pressure Freezing (HPF)-Transmission Electron Microscopy (TEM)

*Candida* spp. yeast cells were grown in YPD at 30°C for 6 h and HPF was carried out as described previously (Walker et al., 2010) with the following modifications. Briefly, samples were prepared by high-pressure freezing with an EMPACT2 high-pressure freezer and rapid transport system (Leica Microsystems Ltd., Milton Keynes, United Kingdom). After freezing, cells were freeze-substituted in substitution reagent (1% [wt/vol] OsO_4_ in acetone) with a Leica EMAFS2. Samples were then embedded in Epoxy resin and additional infiltration was provided under a vacuum at 60°C before embedding in Leica FSP specimen containers and polymerizing at 60°C for 48 h. Semithin survey sections, 0.5 μm thick, were stained with 1% toluidine blue to identify areas containing cells. Ultrathin sections (60 nm) were prepared with a Diatome diamond knife on a Leica UC6 ultramicrotome and stained with uranyl acetate and lead citrate for examination with a Philips CM10 transmission microscope (FEI UK Ltd., Cambridge, United Kingdom) and imaging with a Gatan Bioscan 792 (Gatan United Kingdom, Abingdon, United Kingdom). Image J was used to measure the thickness of the inner (chitin and glucan) and outer cell wall by averaging measurements for 30 cells, for each condition.

### Antifungal Susceptibility Testing

CSF (Merck Research Laboratories, New Jersey, USA) minimum inhibitory concentrations were determined by broth micro-dilution testing using the CLSI guidelines M27-A3. Drug concentrations ranged from 0.016 to 16 μg/ml CSF. Exponentially grown cultures were diluted to 2 × 10^6^ cells/ml in 2x RPMI-1640 and 100 μl of culture was added to each well. Plates were incubated for 24 h at 37°C. After incubation, optical densities were read in a VERSAmax tunable microplate reader (Molecular Devices, California, USA) at 405 nm.

### HPLC Analysis of Cell Wall Composition

Cell walls were extracted as described previously (Mora-Montes et al., 2011). Briefly, each *Candida* species was grown in RPMI-1640 for 3 h at 37°C with shaking at 200 rpm. Each *Candida* species was then subsequently treated with a sub-MIC concentration of CSF (as determined from Table 1) for an additional 2 h, or grown without addition of drug for a further 2 h, as an untreated control. Cells were collected by centrifugation at 4,000 g for 5 min, washed once with chilled deionized water, resuspended in deionized water, and physically fractured with glass beads in a FastPrep machine (Qbiogene). The lysed cells were collected by centrifugation at 4,000 g for 5 min. The pellet containing the cell debris and walls was washed five times with 1 M NaCl, resuspended in extraction buffer (500 mM Tris-HCl buffer, pH 7.5, 2% [wt/vol] SDS, 0.3 M β-mercaptoethanol, and 1 mM EDTA), boiled at 100°C for 10 min, and freeze-dried. To quantify glucan, mannan, and chitin the cell walls were acid hydrolyzed as previously described (Lee et al., 2012). The hydrolyzed samples were analyzed by high-performance anion exchange chromatography with pulsed amperometric detection (HPAEC-PAD) in a carbohydrate analyzer system from Dionex (Surrey, United Kingdom) as described previously (Plaine et al., 2008). The total concentration of each cell wall component was expressed as μg per mg of dried cell wall which was determined by calibration from the standard curves of glucosamine, glucose, and mannose monomers, and converted to a percentage of the total cell wall.

### Proteomic Analysis

Analysis of cell wall proteins was as described previously (Dutton et al., 2014). *Candida* species were grown with or without CSF as described for the HPLC analysis above. Briefly, 2 mg of freeze dried cell wall was mixed with 0.5 M ammonium bicarbonate in water containing 3 mM dithiothreitol and heated at 60°C for 20 min. Iodoacetamide (30 μl of 55 mM stock solution) was then added, and the suspension was incubated in the dark, at 25°C for 10 min. Following addition of 30 μl of 20 mg/ml trypsin, the suspension was incubated at 37°C for 14 h and centrifuged at 14,000 × g for 10 min. The supernatant was freeze-dried and extracted with 10% formic acid, and peptides were purified using ZipTip mC18 pipette tips (Millipore) and dissolved in 0.1% formic acid.

Samples (3 μl) were injected into an LC-MS system which comprised an UltiMate 3000 LC instrument (Dionex Ltd., United Kingdom) fitted with a PepSwift monolithic poly(styrene-codivinylbenzene) (PS-DVB) column (200 μm inside diameter [i.d.] by 5 cm; Dionex) coupled to an HCTultra ion trap mass spectrometer (Bruker Daltonik GmbH, Bremen, Germany) fitted with a low-flow nebulizer in the electrospray ionization (ESI) source and controlled by HyStar software (version 4.0; Bruker Daltonik). Peptides were separated at a flow rate of 2 μl/min using a linear gradient of 0–40% acetonitrile-water-formic acid (80:20:0.04) (solvent B) in water-acetonitrile-formic acid (97:3:0.05) (solvent A) over 40 min, followed by a 1 min column wash in 90% solvent B and a 12-min equilibration step in solvent A. MS/MS data (scan range, *m*/*z* 100–2,200; averages 2) were acquired in positive data-dependent AutoMS(n) mode using the esquireControl software program (version 6.2; Bruker Daltonik). Up to three precursor ions were selected from the MS scan (range, *m*/*z* 300–1,500; averages 3) in each AutoMS(n) cycle. Precursors were actively excluded after being selected twice within a 1-min window, and singly charged ions were also excluded. Peptide peaks were detected (maximum of 9,999 compounds above an intensity threshold of 50,000) and deconvoluted automatically using Data Analysis software (version 3.4; Bruker Daltonik). Mass lists in the form of Mascot Generic Format (^*^. mgf) files were created automatically and used as inputs to Mascot MS/MS ion searches via a local Mascot server (version 2.2; Matrix Science, London, United Kingdom) with a database built from sequence files available from Candida Genome Database (32). The Search parameters used were the following: enzyme = trypsin; fixed modifications = carbamidomethyl (C); variable modifications = oxidation (M); mass values = monoisotopic; peptide mass tolerance = 1.5 Da; fragment mass tolerance = 0.5 Da; max missed cleavages = 1; instrument type = ESI-TRAP. Search results were displayed by Mascot after selection of the following parameters: standard scoring; require bold red; ion score or expect cutoff = 0.05.

### Statistical Analyses

Results from independent replicate experiments are expressed as means ± SD. One-way ANOVA with a Dunnett's *post-hoc* test was used for statistical analysis. Significance was determined as *p* < 0.05.

## Results

### IC_50_ of *Candida* spp to Caspofungin

The susceptibility of each of the sequenced *Candida* spp. strains, used in this study, to CSF was determined following the CLSI-M27-A3 guidelines. *C. albicans, C. dublinensis, C. glabrata*, and *C. tropicalis* were the most susceptible to CSF (Table 1). The IC_50_ range for these susceptible species was between 0.032 and 0.064 μg/ml CSF (Table 1). *C. lusitaniae* and *C. parapsilosis* had CSF IC_50_ measurements of 0.5 and 1 μg/ml, respectively, which was classified as intermediate susceptibility and *C. guilliermondii* was resistant to CSF (Table 1). Therefore, the order of susceptibility to CSF from most susceptible to most resistant of the isolates of the *Candida* spp. tested here was: *C. albicans* > *C. dubliniensis* > *C. glabrata* > *C. tropicalis* > *C. lusitaniae* > *C. parapsilosis* > *C. guilliermondii*.

### *Candida* Species Have Differences in the Ultrastructure of Their Cell Wall

**Figure 1:**
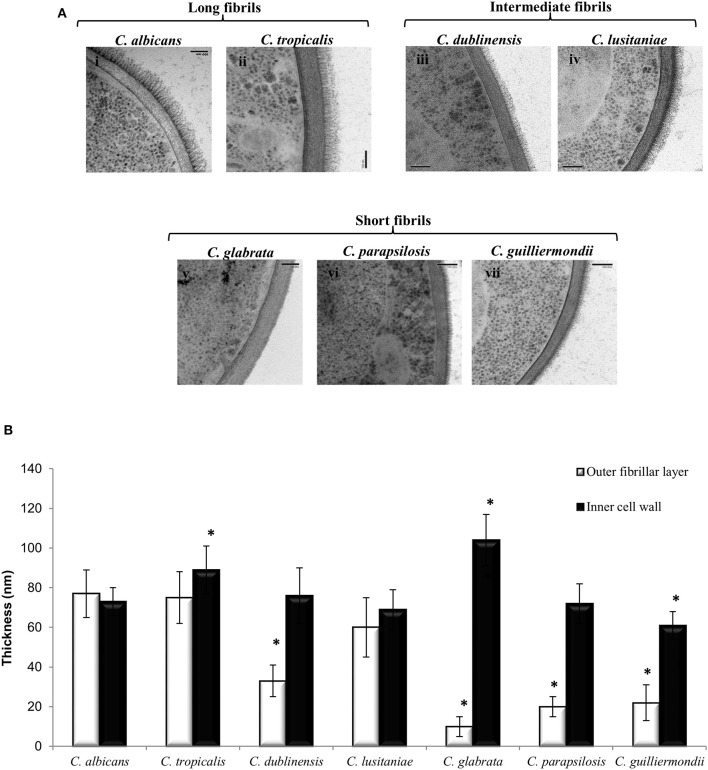
The ultrastructure of the cell wall varies between *Candida* species. **(A)** TEMs of the cell wall of different *Candida* species: *C. albicans* (i)*, C. tropicalis* (ii), *C. dublinensis* (iii), *C. lusitaniae* (iv), *C. glabrata* (v), *C. parapsilosis* (vi) and *C. guilliermondii* (vii). Scale bars are 100 nm. **(B)** The thickness of the inner cell wall (glucan and chitin) and outer fibrillar layer was determined from TEM pictures. Measurements from 30 cells that were sectioned so that cell wall thickness was even all around the cell periphery. Significant differences (*p < 0.05) compared to *C. albicans*. Error bars are SD (*n* = 30).

Ultrastructural differences in the cell wall of each *Candida* species were investigated using high pressure freezing transmission electron microscopy (TEM) which conserves the fungal cell wall architecture. TEM analysis revealed that the inner core of the cell wall of *C. glabrata* (Figure 1A, v) and *C. tropicalis* (Figure 1A, ii) isolates was significantly thicker than the inner core of *C. albicans* isolate (Figure 1A, i; Figure 1B). In contrast to this the inner core of the *C. guilliermondii* cell wall (Figure 1A, vii) was significantly thinner, in comparison to *C. albicans* (Figure 1A, i; Figure 1B). Overall, the largest difference in the cell wall between the *Candida* species was in the outer fibrillar layer (Figure 1B). The variations in length of the outer fibrillar layer were classified as either long (>65 nm), intermediate (30–65 nm), or short (<30 nm) (Figure 1B). *C. albicans* and *C. tropicalis* had the longest outer fibrillar layers, followed by *C. dubliniensis* and *C. lusitaniae* which were classified as having intermediate fibril length (Figure 1B). *C. glabrata, C. parapsilosis*, and *C. guilliermondii* isolates had short outer fibrils on the cell surface, in comparison to the *C. albicans* isolate (Figure 1B).

### *Candida* Species Have Species Specific GPI-Anchored Proteins Present on Their Cell Surface

To determine the differences in the cell wall proteome between the different *Candida* species, LC-MS/MS was used to identify which covalently attached, predicted GPI-modified proteins were present on the surface of each *Candida* species when they were cultured in RPMI-1640 medium (Table 2). Here we have annotated the proteins according to their *C. albicans* orthologs based on sequence similarity according to Candida Genome Database (Skrzypek et al., 2016). A core set of predicted cell wall proteins were identified in all species (Table 2). The hyphal specific protein, Hyr1, was uniquely detected in the cell wall of *C. albicans*, commensurate with it being a *C. albicans*-specific hyphal-associated protein and *C. albicans* will undergo filamentation in RPMI-1640 medium. GPI-anchored proteins that were identified specifically in the cell wall of *C. dubliniensis* included two Als proteins involved in adhesion and the secreted aspartyl protease, Sap9 (Table 2). A fungal-specific phospholipase and Pga59 orthologs were detected only on the cell surface of *C. lusitaniae* (Table 2). The cell wall of *C. parapsilosis* was found to contain orthologs of the yeast wall protein, Ywp1/Pga24, and Pga30, a protein of unknown function (Table 2).

**Table 2:** *Candida* species-specific detection of cell wall GPI-anchored proteins by LC/MS/MS proteomics.

**Species specific detection of putative GPI-anchored proteins^a^^,^^b^^,^^c^**						
***C. albicans***	***C. dubliniensis***	***C. lusitaniae***	***C. parapsilosis***	***C. tropicalis***	***C. glabrata***	***C. guilliermondii***
Hyr1 (C1_13450W_A)	Als4 (CD36_64610)	Pga31 (CLUG_00164)	Pga24/Ywp1 (CPAR2_806670)	Pga24/Ywp1 (CTRG_01856)	Epa6 (CAGL0C00110g)	Pga24/Ywp1 (DEHA2A14366g)
	Als2 (CD36_64800)	Rbt1 (CLUG_03306)	Pga30 (CPAR2_402000)		Epa3 (CAGL0E06688g)	
	Sap9 (CD36_83850)				Awp4 (CAGL0J12001g)	

a *Proteins assigned common names according to their C. albicans orthologs (Butler et al., 2009). Unique protein IDs in brackets*.

b *Detected in at least one of three biological replicate samples*.

c *In addition the following proteins were detected in all samples: Ssr1 (C7_00860W_A), Pga4 (C5_05390C_A), Crh11 (C4_02900C_A)*.

Caspofungin treatment has been previously shown to alter cell wall architecture and increases chitin production in *C. albicans, C. tropicalis, C. krusei, C. parapsilosis*, and *C. guilliermondii* (9). The effect of CSF treatment on the cell wall proteome of each *Candida* species was also investigated by treating each *Candida* spp. with their IC_50_ concentration of caspofungin (Table 3). Certain proteins were detected only at the cell surface in response to CSF treatment in addition to the proteins listed that are found on the surface of untreated cells. The proteins expressed in response to caspofungin varied between the different *Candida* spp. Orthologs of the transglycosidase, Utr2, were detected on the cell surface of the majority of *Candida* species in response to CSF treatment with the exception of *C. glabrata* (Table 3). Orthologs of Pga31, a GPI-anchored protein of unknown function was detected on the surface of the species that were most sensitive to CSF, such as *C. albicans, C. dublinensis*, and *C. tropicalis* in response to CSF treatment (Table 3). A protein related to Pga30, a member of the same family as Pga31 was detected in the cell wall of *C. guilliermondii* treated with CSF. *C. lusitaniae* and *C. parapsilosis*, which have intermediate susceptibility to CSF, were found to have Als-like proteins on their cell surface in response to CSF treatment (Table 3).

**Table 3:** Caspofungin treatment specific proteins in different *Candida* species in RPMI-1640 medium.

**Species**	**Cell wall proteins specific to CSF-treated cells^a^^,^^b^^,^^c^**				
*C. albicans*	Utr2 (C3_01730C_A)	Pga31 (C4_04080C_A)	Ecm33 (C1_03190C_A)	Rbt1 (C4_03520C_A)	Pga29 (C4_04050C_A)
*C. dubliniensis*	Utr2 (CD36_81610)	Pga31 (CD36_43780)	Phr2 (CD36_00220)		
*C. lusitaniae*	Utr2 (CLUG_02005)	Pga62 (CLUG_00242)	Als1	Phr1 (CLUG_01043)	Pga24 (CLUG_00242)
*C. parapsilosis*	Utr2 (CPAR2_503190)		Als1		
*C. tropicalis*	Utr2 (CTRG_02140)	Pga31 (CTRG_00350)	Ecm33 (CTRG_00105)		
*C. glabrata*	Pir3 (CAGL0M08492g)	Crh1 (CAGL0G09449g)			
*C. guilliermondii*	Utr2 (PGUG_00573)	Pga30 (PGUG_01942)	Plb5 (PGUG_01288)		

a *Proteins assigned common names according to their C. albicans orthologs (Butler et al., 2009). Unique protein IDs in brackets*.

b *Detected in at least one of three biological replicate samples*.

c *These proteins were not detected in the cell walls of untreated cultures*.

### Cell Wall Composition of Different *Candida* Species in the Presence or Absence of CSF

The carbohydrate composition of the cell walls of the different *Candida* spp., in the presence or absence of an IC_50_ concentration of caspofungin, was determined by acid hydrolysis of isolated cell walls and analysis by high-performance liquid chromatography. *C. tropicalis* had substantially higher chitin content than *C. albicans*, whereas *C. glabrata* and *C. guilliermondii* had significantly less chitin compared to *C. albicans* (Table 4). The majority of *Candida* spp. had comparable levels of glucan in the cell wall, with the exception of *C. tropicalis*, which had significantly reduced glucan compared to *C. albicans* and *C. glabrata*, which had a higher glucan content (Table 4). The quantity of mannan in the cell wall was significantly reduced in *C. glabrata, C. parapsilosis*, and *C. guilliermondii* compared to *C. albicans* (Table 4) in agreement with the TEM analysis which suggested these species have shorter mannan fibrils on their outer surfaces. In most cases treatment with CSF resulted in an increase in chitin content, with the exception of *C. glabrata* and *C. parapsilosis* (Table 4). As expected all *Candida* spp. isolates tested had a reduction in β-1,3-glucan in response to CSF treatment. Exposure to an IC_50_ concentration of CSF led to a significant increase in mannan content in all *Candida* spp., with the exception of *C. tropicalis* and *C. dublinensis* isolates (Table 4).

**Table 4:** Quantification of the components of the cell wall of *Candida* species, with and without caspofungin treatment ^a^^,^^b^^,^^c^^*^^#^.

***Candida* spp**.	**% Dried cell wall**		
	**Chitin**	**Glucan**	**Mannan**
*C. albicans*	4 ± 0.2 (100%)	66 ± 3 (100%)	30 ± 2 (100%)
*C. albicans +* CSF^c^	8 ± 1^#^ (200%)	43 ± 5^#^ (65%)	49 ± 3^#^ (163%)
*C. tropicalis*	5 ± 0.5^*^ (100%)	54 ± 2^*^ (100%)	41 ± 6 (100%)
*C. tropicalis* + CSF	12 ± 4 (240%)^#^	40 ± 7 (74%)^#^	49 ± 2 (120%)
*C. dubliniensis*	5 ± 0.8 (100%)	63 ± 4 (100%)	32 ± 1 (100%)
*C. dubliniensis* + CSF	13 ± 2^#^ (260%)	49 ± 6^#^ (78%)	38 ± 3 (119%)
*C. lusitaniae*	4 ± 0.1 (100%)	70 ± 8 (100%)	26 ± 2 (100%)
*C. lusitaniae* + CSF	9 ± 3^#^ (225%)	53 ± 11^#^ (76%)	38 ± 3^#^ (146%)
*C. glabrata*	2 ± 0.1^*^ (100%)	81 ± 5^*^ (100%)	17 ± 4^*^ (100%)
*C. glabrata* + CSF	1 ± 0.2 (50%)	67 ± 3^#^ (83%)	32 ± 2^#^ (188%)
*C. parapsilosis*	7 ± 5 (100%)	75 ± 4 (100%)	18 ± 3^*^ (100%)
*C. parapsilosis* +CSF	8 ± 3 (114%)	62 ± 2^#^ (83%)	30 ± 6^#^ (167%)
*C. guilliermondii*	2 ± 1^*^ (100%)	79 ± 6 (100%)	19 ± 2^*^ (100%)
*C. guilliermondii +* CSF	6 ± 0.5^#^ (300%)	59 ± 8^#^ (75%)	36 ± 5^#^ (189%)

a *Cell walls were acid hydrolyzed and released monosaccharide was detected*.

b *Results are expressed as a % of dried cell wall of the untreated data ± SD. n = 3 biological replicates. For + CSF samples % in brackets is relative to untreated samples which were set as 100%*.

c *IC_50_ concentration of CSF was used for each species*.

* *Significantly different compared to untreated cells of C. albicans (P = 0.05)*.

# *Significantly different compared to untreated cells of the same species (P = 0.05)*.

### *Candida* Species Have Differences in Surface Exposure of Chitin and Glucan in Response to CSF Treatment

Exposure of chitin and glucan on the cell surface is known to influence the immune recognition of fungal cells (Wheeler and Fink, 2006; Wheeler et al., 2008; Mora-Montes et al., 2011). Fluorescent staining was used to determine whether the differences in outer fibril length between the *Candida* species isolates correlated with variations in exposure of chitin and β-1,3-glucan on the cell surface. The exposure of chitin was determined using 25 μg/ml Wheat Germ Agglutinin-Texas Red (WGA-TR) and exposed β-1,3-glucan was stained with 5 μg/ml Fc:Dectin1- Alexa 488 (Table 5, Supplementary Figure 1). The exposure of chitin and β-1,3-glucan, after treatment with an IC_50_ concentration of CSF for 6 h, was determined for each *Candida* species isolate. Exposure of β-1,3-glucan was significantly higher on the surface of untreated *C. glabrata* and *C. guilliermondii* compared to *C. albicans* (Table 5). Treatment with CSF, at the IC_50_, resulted in increased exposure of β-1,3-glucan on the surface of *C. albicans, C. tropicalis, C. dubliniensis, C. lusitaniae*, and *C. guilliermondii* (Table 5). However, CSF treatment had no effect on the exposure of β-1,3-glucan of *C. glabrata* and *C. parapsilosis* (Table 5). Measurement of exposed chitin on the cell surface determined that the *C. glabrata* isolate had less chitin exposed on the outer surface compared to *C. albicans* isolate (Table 5). Treatment of *C. albicans, C. tropicalis, C. dubliniensis, C. lusitaniae*, and *C. guilliermondii* with CSF resulted in increased detection of chitin on the cell surface (Table 5). The exception to this was *C. glabrata* and *C. parapsilosis* which had no change in exposure of chitin, in response to CSF treatment (Table 5).

**Table 5:** Quantification of the exposure of β-1,3-glucan and chitin of *Candida* species, with and without caspofungin treatment^a^.

***Candida* species**	**Glucan exposure (mean fluorescence intensity)**		**Chitin exposure (mean fluorescence intensity)**	
	**No treatment**	**Caspofungin treatment**	**No treatment**	**Caspofungin treatment**
*C. albicans*	179 ± 20	450 ± 34^#^	167 ± 17	488 ± 51^#^
*C. dubliniensis*	211 ± 14	374 ± 52^#^	160 ± 26	404 ± 40^#^
*C. glabrata*	273 ± 21*	257 ± 39	89 ± 18*	79 ± 20
*C. tropicalis*	185 ± 41	607 ± 66^#^	178 ± 22	516 ± 54^#^
*C. lusitaniae*	171 ± 29	468 ± 49^#^	159 ± 31	438 ± 35^#^
*C. parapsilosis*	201 ± 13	189 ± 30	172 ± 28	202 ± 45
*C. guilliermondii*	263 ± 36*	537 ± 72^#^	160 ± 29	445 ± 49^#^

a The average relative β-1,3-glucan and chitin contents of individual cells from different Candida species were determined by measuring the intensity of Fc:Dectin1–Alexa488 and WGA-Texas Red fluorescence. Measurements were made on untreated control cultures and after growth with caspofungin at the specific IC_50_ for each species (as determined in Table 1). Statistical differences are shown for comparison to untreated cells of C. albicans (*P = 0.05) or untreated cells of the same species (^#^P = 0.05), and data are means with standard deviations (n = 50).

### Relationship Between Phagocytosis and Exposure of Chitin and Glucan

**Figure 2:**
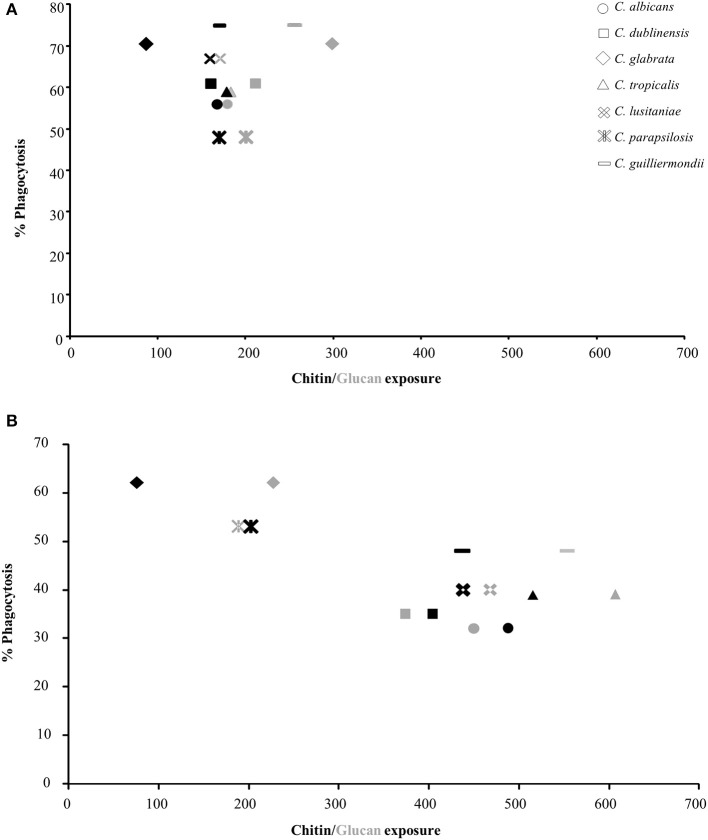
Correlation between exposure of chitin and glucan on the cell surface of *Candida* species and phagocytosis by J774 macrophages. The values for exposure of chitin and β-1,3-glucan were taken from Table 5. Phagocytosis of *Candida* spp. by macrophages was determined by fluorescence microscopy. All cells were stained with 10 μg/ml FITC prior to exposure to macrophages. FITC-stained cells were exposed to J774 macrophages for 1 h, after which non-phagocytosed cells were stained with 25 μg/ml CFW. The number of macrophages which had phagocytosed *Candida* cells were then counted (*n* = 300). **(A)** Untreated *Candida* spp. cells. **(B)** CSF treated *Candida* cells. Black shapes = chitin (WGA-TR), gray shapes = glucan (Fc:dectin1-alexa488).

To determine the effect of exposure of chitin and glucan on phagocytosis, the uptake of isolates of each *Candida* species by J774 macrophages was determined. The yeasts were cultured with and without sub-MIC CSF treatment and phagocytosis correlated to the exposure of chitin and β-1,3-glucan on the cell surface (Figure 2, Supplementary Table 1). The majority of *Candida* species had low levels of chitin and β-1,3-glucan exposed naturally on the cell surface, under the growth conditions used (Figure 2A). The exception was *C. glabrata* and *C. guilliermondii* which had significantly more β-1,3-glucan exposed on the cell surface, compared to *C. albicans*, which correlated with these *Candida* species having the highest phagocytosis by J774 macrophages (Figure 2A). To determine the effect of altered exposure of β-1,3-glucan and chitin, the phagocytosis of CSF-treated cells was also measured (Figure 2B). In all cases the combined increase in exposure of chitin and β-1,3-glucan, in CSF-treated cells, resulted in a significant decrease in phagocytosis by macrophages (Figure 2B). Treatment of *C. glabrata* and *C. parapsilosis* with CSF had little effect on the exposure of the outer cell wall components and consequently there was no significant change in their phagocytosis by macrophages (Figure 2B). Pearson Correlation analysis demonstrated that there was a positive correlation between glucan exposure and chitin exposure and caspofungin treatment [*r*_(12)_ = 0.703, *p* = 0.005; *r*_(12)_ = 0.699, *p* = 0.005 respectively] but a negative correlation between % phagocytosis and caspofungin treatment [*r*_(12)_ = −0.763, *p* = 0.002].

### Cytokine Production Varies Between *Candida* Species and Is Reduced in Response to CSF Treatment

**Figure 3:**
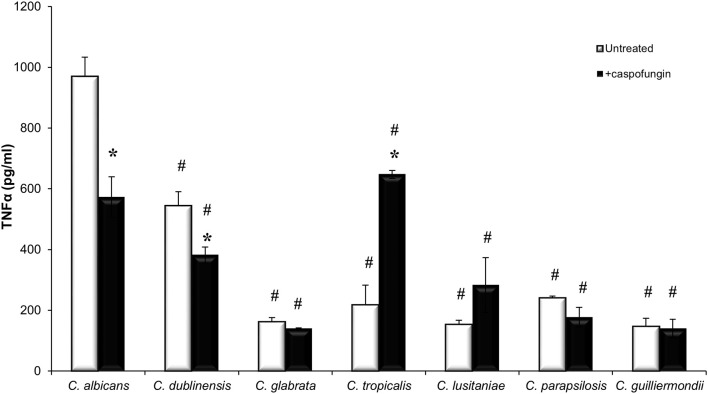
*Candida* species stimulate different immune responses. Live cells of each *Candida* species, which had been grown with or without an IC_50_ concentration of caspofungin for 6 h, were exposed to J774 macrophages for 4 h. After 4 h of co-incubation, supernatants were collected and cytokine production was measured. Significant differences (^#^*p* < 0.05) compared to *C. albicans*. Significant differences (*p < 0.05) compared to untreated cells of the same species. Error bars are SD (*n* = 3).

Increased exposure of β-1,3-glucan has previously been shown to stimulate cytokine production whereas increased exposure of chitin has been shown to result in a decrease in cytokine production (Wheeler and Fink, 2006; Wheeler et al., 2008; Mora-Montes et al., 2011). To determine the cytokine production stimulated by the different *Candida* species, TNFα production was measured when untreated live cells of each *Candida* species were exposed to J774 macrophages for 4 h (Figure 3). Live cells of *C. albicans* and *C. dubliniensis* stimulated the highest levels of TNFα production (Figure 3). The other *Candida* species all stimulated very low levels of TNFα (Figure 3), which remained unchanged even after 24 h (data not shown). Treatment with an IC_50_ concentration of CSF, specific to each *Candida* species, led to a significant decrease in TNFα production in *C. albicans* and *C. dubliniensis*, compared to untreated cells (Figure 3). Treatment with CSF had no effect on TNFα production in the other *Candida* species isolates, with the exception of the *C. tropicalis* isolate which had a significant increase in TNFα production compared to untreated cells (Figure 3). *C. tropicalis* also had the highest level of surface-exposed β(1,3)-glucan when treated with CSF.

## Discussion

This study aimed to directly compare the structure and composition of the cell walls of different *Candida* species, examine how the cell wall was altered in response to caspofungin treatment and assess how this impacted on fungal: murine macrophage interactions. We demonstrated that the length of the outer fibrillar layer varied between the species isolates tested and that, in most cases, reduced fibril length correlated with increased exposure of β-1,3-glucan on the cell surface. *C. glabrata* and *C. guilliermondii*, which had naturally more β-1,3-glucan exposed on the cell surface, were phagocytosed more efficiently by J774 macrophages. This is in agreement with a previous study that showed that in a mixed population of *C. albicans* and *C. glabrata* cells, *C. glabrata* is preferentially phagocytosed over *C. albicans* (Keppler-Ross et al., 2010). Similar to our observations, Estrada-Mata et al. (2016) also demonstrated that *C. parapsilosis* had less mannan content but more β-1,3-glucan exposed on the surface compared to *C. albicans* which correlated with an increase in cytokine production by human PBMCs (Estrada-Mata et al., 2016). Dectin-1 has been shown to play an important role in recognition of *C. parapsilosis* by human PBMCs (Toth et al., 2013). In our study *C. parapsilosis* induced little cytokine production from J774 macrophages at 4 h, but differences in the source of immune cells and the time that *Candida* cells were exposed to immune cells before performing the ELISAs may account for these differences.

There were also notable differences in the cell wall proteins that reside on the surface of the different *Candida* species, for example, the GPI-anchored proteins, Epa3, Epa6, and Awp4, were identified specifically on the cell wall of *C. glabrata* under the growth conditions tested. These are known to be adhesion proteins in *C. glabrata* which are associated with biofilm formation (de Groot et al., 2008; Kraneveld et al., 2011). The presence of cell wall proteins on the cell surface of *C. glabrata, C. parapsilosis*, and *C. tropicalis* has been shown to be dependent on the growth conditions tested (Karkowska-Kuleta et al., 2019).

Treatment with CSF is known to result in a compensatory increase in cell wall chitin levels and result in increased exposure of chitin and β-1,3-glucan on the surface of *C. albicans* cells (Wheeler and Fink, 2006; Walker et al., 2008, 2013; Wheeler et al., 2008; Mora-Montes et al., 2011). All of the *Candida* species isolates tested, with the exception of *C. glabrata* and *C. parapsilosis* isolates, demonstrated a compensatory increase in cell wall chitin content in response to CSF treatment. Likewise, treatment with CSF also resulted in an increase in exposure of chitin and β-1,3-glucan on the cell surface for the majority of species tested. Notable exceptions were *C. glabrata* and *C. parapsilosis* with no changes in glucan exposure in response to caspofungin treatment. This increase in exposure of the inner cell wall polysaccharides, did not increase the number of macrophages that had engulfed the fungal cells in fact caspofungin treatment resulted in a reduction in % phagocytosis. The majority of live *Candida* spp. cells stimulated low levels of TNFα production. In comparison the *C. albicans* and *C. dublinensis* isolates stimulated elevated levels of TNFα but these levels were significantly reduced when macrophages were exposed to the same isolates after caspofungin treatment. A notable exception to this trend was CSF-treated *C. tropicalis* isolate which stimulated significantly elevated TNFα production compared to untreated cells attaining a level comparable to *C. albicans* and *C. dublinensis*. *C. tropicalis* had the highest level of Fc-dectin1-Alex488 staining with and without caspofungin treatment. These findings suggested that the immune stimulatory properties of the different *Candida* species is not solely dependent upon β-1,3-glucan exposure and recognition by Dectin-1 and other factors may come into play (Wagener et al., 2014). Recently, treatment with either fragments of chitin or β-1,3-glucan from *Aspergillus fumigatus* were shown to stimulate production of TNFα. In addition, combined treatment with both chitin and β-1,3-glucan resulted in a synergistic increase in TNFα production (Dubey et al., 2014). This is in contrast with the findings here where other changes at the cell surface including increased chitin exposure appeared to override any stimulation of the immune response elicited by glucan exposure. The differences in stimulation of the immune response may be due to variations in size of the cell wall polysaccharides and the way the polymer is presented to immune cells. Small particles of chitin are known to illicit a greater cytokine response compared to larger chitin fragments (Da Silva et al., 2009). Likewise, differences in polysaccharide structure may influence the immune response. The chitin microfibrils of *A. fumigatus* and *C. albicans* may have a different architecture, which could influence the immune response (Gow and Gooday, 1983; Lenardon et al., 2007). Recently, hyphal β-1,3-glucan of *C. albicans* was found to have a unique cyclical structure that is absent in β-1,3-glucan from the yeast form (Lowman et al., 2014). Compared to β-1,3-glucan from yeast cells, hyphal glucan was shown to induce an immune response in macrophages and human peripheral blood mononuclear cells through a Dectin-1-dependent mechanism. In addition CSF treatment has also been shown to not only effect β-1,3-glucan exposure but also its nano-structure with the density of dectin-1 binding sites increasing leading to increased phagocytosis by dendritic cells (Lin et al., 2016). To dissect further whether glucan and/or chitin plays the main role in caspofungin-mediated dampening of the immune response, it would be interesting to evaluate the effect of blocking receptors of both polysaccharides on interaction with macrophages. Another consideration is that the change in GPI-modified proteins on the cell surface, in response to CSF treatment, may also influence the interaction with immune cells. For example, the transglycosidase, Utr2, was detected on the surface of the majority of *Candida* species in response to CSF treatment. Utr2 is involved in crosslinking chitin and β-1,3-glucan and forms part of a compensatory mechanism activated in response to cell weakening induced by echinocandin treatment (Pardini et al., 2006). A *C. albicans utr2*Δ mutant was found to have the same susceptibility to micafungin as the wild type (Alberti-Segui et al., 2004). Deletion of *UTR2* in *C. albicans* resulted in attenuated virulence in a mouse model of systemic candidiasis. Despite this attenuation in virulence the fungal burdens from the kidneys of mice infected with the *utr2*Δ mutant were comparable to that of mice infected with wild-type *C. albicans*. This suggests that in the absence of *UTR2*, cells do not elicit the same inflammatory response as wild-type cells and enables the *utr2*Δ mutant to be tolerated by the host, leading to survival of the infected mice (Pardini et al., 2006). Therefore, the increased abundance of Utr2 on the cell surface in response to caspofungin treatment may also contribute to altered interactions with host cells.

*Candida guilliermondii* and *C. parapsilosis* are known to be intrinsically less sensitive to the echinocandins. Both species had thin walls and their cell wall proteomes differed from CSF-susceptible species. Pga24/Ywp1 orthologs were detected on their cell surfaces when grown on RPMI-1640 medium. In *C. albicans* Pga24 is associated with the yeast cell wall (Granger et al., 2005) and here was not detected when *C. albicans* was grown on RPMI-1640 medium. Mutation of *PGA24* did not alter echinocandin susceptibility of *C. albicans* (Plaine et al., 2008). The role of this surface protein in echinocandin susceptibility of *C. guilliermondii* and *C. parapsilosis* remains to be determined.

The ability of live cells of each isolate of the different *Candida* species to stimulate cytokine production varied significantly (Figure 3). *C. albicans* and *C. dubliniensis* isolates, tested here, elicited the highest production of TNFα. In contrast to this the other isolates representing the different *Candida* species stimulated negligible levels of TNFα when exposed to J774 macrophages for 4 h. Previous work has demonstrated that *C. parapsilosis, C. tropicalis, C. lusitaniae*, and *C. guilliermondii* in addition to *C. albicans* and *C. dubliniensis*, stimulated production of TNFα from murine peritoneal macrophages and human PBMCs after 24 h (Aybay and Imir, 1996; Navarro-Arias et al., 2019). The most likely reason for the difference observed at the 4 h time point is that *C. albicans* and *C. dubliniensis* are the only two species which form true hyphae and were capable of germinating within macrophages, and consequently escaping from macrophages, faster than any of the other *Candida* species. For example, *C. glabrata* yeast cells have been shown to replicate within macrophages for up to 3 days before causing lysis of the macrophage (Seider et al., 2011). Therefore, the low levels of cytokine production that have been observed as a result of exposure of macrophages to live *C. glabrata* is thought to be due to *C. glabrata* evading host defenses by hiding within immune cells (Seider et al., 2011, 2014; Brunke et al., 2014). However, it is possible for *C. glabrata* to filament under certain conditions. Prolonged exposure of *C. glabrata* to macrophages led to filamentous growth which was a result of a point mutation in *CHS2* (Lewis et al., 2013). Treatment of *C. albicans* and *C. dubliniensis* with CSF resulted in a decrease in production of TNFα and reduced phagocytosis by macrophages. Exposure of *C. albicans* to caspofungin has previously been shown to reduce cytokine production in both live and heat killed *C. albicans* cells, as a result of increased exposure of chitin on the cell surface (Mora-Montes et al., 2011).

Here we examined caspofungin induced changes at the cell surface of representative isolates of different *Candida* species. We detected drug-induced changes in the repertoire of covalently attached surface proteins and alterations in the exposure of cell wall carbohydrates of contributing to their recognition by J774 macrophages. The cell surface is highly variable both between and within species and so these investigations should be expanded to a wider range of isolates of the different species to determine the extent of cell surface variability and how it impacts on host interactions and drug susceptibility.

## Data Availability Statement

The raw data supporting the conclusions of this article will be made available by the authors, without undue reservation, to any qualified researcher.

## Author Contributions

LW and CM contributed to acquisition, analysis, and interpretation of data for the work. All authors contributed to the design of the work and approved the submitted and final version.

## Conflict of Interest

The authors declare that the research was conducted in the absence of any commercial or financial relationships that could be construed as a potential conflict of interest.

## References

[B1] Alberti-Segui C. Morales A. J. Xing H. Kessler M. M. Willins D. A. Weinstock K. G.. (2004). Identification of potential cell-surface proteins in *Candida albicans* and investigation of the role of a putative cell-surface glycosidase in adhesion and virulence. Yeast 21, 285–302. 10.1002/yea.1061 15042589

[B2] Alexander B. D. Johnson M. D. Pfeiffer C. D. Jimenez-Ortigosa C. Catania J. Booker R.. (2013). Increasing echinocandin resistance in *Candida glabrata*: clinical failure correlates with presence of *FKS* mutations and elevated minimum inhibitory concentrations. Clin. Infect. Dis. 56, 1724–1732. 10.1093/cid/cit136 23487382 PMC3658363

[B3] Aybay C. Imir T. (1996). Tumor necrosis factor (TNF) induction from monocyte/macrophages by *Candida* species. Immunobiology 196, 363–374. 10.1016/S0171-2985(96)80059-3 9061377

[B4] Balashov S. V. Park S. Perlin D. S. (2006). Assessing resistance to the echinocandin antifungal drug caspofungin in *Candida albicans* by profiling mutations in *FKS1*. Antimicrob. Agents Chemother. 50, 2058–2063. 10.1128/AAC.01653-05 16723566 PMC1479158

[B5] Baltch A. L. Lawrence D. Ritz W. J. Andersen N. Bopp L. H. Michelsen P. B. (2012b). Effects of anidulafungin and voriconazole, singly and in combination, on cytokine/chemokine production by human monocyte-derived macrophages infected with *Candida glabrata* or activated by lipopolysaccharide. Chemotherapy 58, 146–151. 10.1159/000337076 22584412

[B6] Baltch A. L. Lawrence D. A. Ritz W. J. Andersen N. J. Bopp L. H. Michelsen P. B. (2012a). Effects of echinocandins on cytokine/chemokine production by human monocytes activated by infection with *Candida glabrata* or by lipopolysaccharide. Diagn. Microbiol. Infect. Dis. 72, 226–233. 10.1016/j.diagmicrobio.2011.11.004 22209510

[B7] Brown G. D. Gordon S. (2001). Immune recognition. A new receptor for beta-glucans. Nature 413, 36–37. 10.1038/35092620 11544516

[B8] Brunke S. Seider K. Fischer D. Jacobsen I. D. Kasper L. Jablonowski N.. (2014). One small step for a yeast–microevolution within macrophages renders *Candida glabrata* hypervirulent due to a single point mutation. PLoS Pathog. 10:e1004478. 10.1371/journal.ppat.1004478 25356907 PMC4214790

[B9] Butler G. Rasmussen M. D. Lin M. F. Santos M. A. Sakthikumar S. Munro C. A.. (2009). Evolution of pathogenicity and sexual reproduction in eight Candida genomes. Nature. 459, 657–662. 10.1038/nature08064 19465905 PMC2834264

[B10] Da Silva C. A. Chalouni C. Williams A. Hartl D. Lee C. G. Elias J. A. (2009). Chitin is a size-dependent regulator of macrophage TNF and IL-10 production. J. Immunol. 182, 3573–3582. 10.4049/jimmunol.0802113 19265136

[B11] de Groot P. W. Kraneveld E. A. Yin Q. Y. Dekker H. L. Gross U. Crielaard W.. (2008). The cell wall of the human pathogen *Candida glabrata*: differential incorporation of novel adhesin-like wall proteins. Eukaryot. Cell 7, 1951–1964. 10.1128/EC.00284-08 18806209 PMC2583536

[B12] Dubey L. K. Moeller J. B. Schlosser A. Sorensen G. L. Holmskov U. (2014). Induction of innate immunity by *Aspergillus fumigatus* cell wall polysaccharides is enhanced by the composite presentation of chitin and beta-glucan. Immunobiology 219, 179–188. 10.1016/j.imbio.2013.10.003 24286790

[B13] Dutton L. C. Nobbs A. H. Jepson K. Jepson M. A. Vickerman M. M. Aqeel Alawfi S.. (2014). *O*-mannosylation in *Candida albicans* enables development of interkingdom biofilm communities. MBio 5:e00911–14. 10.1128/mBio.00911-14 24736223 PMC3993854

[B14] Estrada-Mata E. Navarro-Arias M. J. Perez-Garcia L. A. Mellado-Mojica E. Lopez M. G. Csonka K.. (2016). Members of the *Candida parapsilosis* complex and *Candida albicans* are differentially recognized by human peripheral blood mononuclear cells. Front. Microbiol. 6:1527. 10.3389/fmicb.2015.01527 26793173 PMC4710749

[B15] Fidan I. Yesilyurt E. Kalkanci A. Aslan S. O. Sahin N. Ogan M. C.. (2014). Immunomodulatory effects of voriconazole and caspofungin on human peripheral blood mononuclear cells stimulated by *Candida albicans* and *Candida krusei*. Am. J. Med. Sci. 348, 219–223. 10.1097/MAJ.0000000000000236 24662309

[B16] Garcia-Effron G. Chua D. J. Tomada J. R. DiPersio J. Perlin D. S. Ghannoum M.. (2010). Novel *FKS* mutations associated with echinocandin resistance in *Candida* species. Antimicrob. Agents Chemother. 54, 2225–2227. 10.1128/AAC.00998-09 20145084 PMC2863628

[B17] Gillum A. M. Tsay E. Y. Kirsch D. R. (1984). Isolation of the *Candida albicans* gene for orotidine-5'-phosphate decarboxylase by complementation of S. cerevisiae ura3 and *E. coli* pyrF mutations. Mol. Gen. Genet. 198, 179–182. 10.1007/bf00328721 6394964

[B18] Gow N. A. Netea M. G. Munro C. A. Ferwerda G. Bates S. Mora-Montes H. M.. (2007). Immune recognition of *Candida albicans* beta-glucan by dectin-1. J. Infect. Dis. 196, 1565–1571. 10.1086/523110 18008237 PMC2655640

[B19] Gow N. A. R. Gooday G. W. (1983). Ultrastructure of chitin in hyphae of *Candida albicans* and other dimorphic and mycelial fungi. Protoplasma 115, 52–58. 10.1007/BF01293580

[B20] Gow N. A. R. Latge J.-P. Munro C. A. (2017). The fungal cell wall: structure, biosynthesis, and function. Microbiol. Spectr. 5, 1–25. 10.1128/microbiolspec.FUNK-0035-2016 28513415 PMC11687499

[B21] Graham L. M. Tsoni S. V. Willment J. A. Williams D. L. Taylor P. R. Gordon S.. (2006). Soluble Dectin-1 as a tool to detect beta-glucans. J. Immunol. Methods 314, 164–169. 10.1016/j.jim.2006.05.013 16844139

[B22] Granger B. L. Flenniken M. L. Davis D. A. Mitchell A. P. Cutler J. E. (2005). Yeast wall protein 1 of *Candida albicans*. Microbiology 151, 1631–1644. 10.1099/mic.0.27663-0 15870471

[B23] Jouault T. Ibata-Ombetta S. Takeuchi O. Trinel P. A. Sacchetti P. Lefebvre P.. (2003). *Candida albicans* phospholipomannan is sensed through toll-like receptors. J. Infect. Dis. 188, 165–172. 10.1086/375784 12825186

[B24] Karkowska-Kuleta J. Satala D. Bochenska O. Rapala-Kozik M. Kozik A. (2019). Moonlighting proteins are variably exposed at the cell surfaces of *Candida glabrata, Candida parapsilosis* and *Candida tropicalis* under certain growth conditions. BMC Microbiol. 19, 145–149. 10.1186/s12866-019-1524-5 31269895 PMC6609379

[B25] Keppler-Ross S. Douglas L. Konopka J. B. Dean N. (2010). Recognition of yeast by murine macrophages requires mannan but not glucan. Eukaryot. Cell 9, 1776–1787. 10.1128/EC.00156-10 20833894 PMC2976302

[B26] Kohatsu L. Hsu D. K. Jegalian A. G. Liu F. T. Baum L. G. (2006). Galectin-3 induces death of *Candida* species expressing specific beta-1,2-linked mannans. J. Immunol. 177, 4718–4726. 10.4049/jimmunol.177.7.4718 16982911

[B27] Kraneveld E. A. de Soet J. J. Deng D. M. Dekker H. L. de Koster C. G. Klis F. M.. (2011). Identification and differential gene expression of adhesin-like wall proteins in *Candida glabrata* biofilms. Mycopathologia 172, 415–427. 10.1007/s11046-011-9446-2 21769633

[B28] Kurtz M. B. Douglas C. M. (1997). Lipopeptide inhibitors of fungal glucan synthase. Med. Mycol. 35, 79–86. 10.1080/02681219780000961 9147267

[B29] Lee K. K. Maccallum D. M. Jacobsen M. D. Walker L. A. Odds F. C. Gow N. A.. (2012). Elevated cell wall chitin in *Candida albicans* confers echinocandin resistance *in vivo*. Antimicrob. Agents Chemother. 56, 208–217. 10.1128/AAC.00683-11 21986821 PMC3256049

[B30] Lenardon M. D. Whitton R. K. Munro C. A. Marshall D. Gow N. A. (2007). Individual chitin synthase enzymes synthesize microfibrils of differing structure at specific locations in the *Candida albicans* cell wall. Mol. Microbiol. 66, 1164–1173. 10.1111/j.1365-2958.2007.05990.x 17971081 PMC2780561

[B31] Lewis L. E. Bain J. M. Okai B. Gow N. A. Erwig L. P. (2013). Live-cell video microscopy of fungal pathogen phagocytosis. J. Vis. Exp. 2013:50196 10.3791/50196 PMC3582652 23329139

[B32] Lin J. Wester M. J. Graus M. S. Lidke K. A. Neumann A. K. (2016). Nanoscopic cell-wall architecture of an immunogenic ligand in *Candida albicans* during antifungal drug treatment. Mol. Biol. Cell 27, 1002–1014. 10.1091/mbc.E15-06-0355 26792838 PMC4791122

[B33] Lowman D. W. Greene R. R. Bearden D. W. Kruppa M. D. Pottier M. Monteiro M. A.. (2014). Novel structural features in *Candida albicans* hyphal glucan provide a basis for differential innate immune recognition of hyphae versus yeast. J. Biol. Chem. 289, 3432–3443. 10.1074/jbc.M113.529131 24344127 PMC3916545

[B34] Marakalala M. J. Vautier S. Potrykus J. Walker L. A. Shepardson K. M. Hopke A.. (2013). Differential adaptation of *Candida albicans in vivo* modulates immune recognition by dectin-1. PLoS Pathog. 9:e1003315. 10.1371/journal.ppat.1003315 23637604 PMC3630191

[B35] Marti-Carrizosa M. Sanchez-Reus F. March F. Canton E. Coll P. (2015). Implication of C*andida parapsilosis FKS1* and *FKS2* mutations in reduced echinocandin susceptibility. Antimicrob. Agents Chemother. 59, 3570–3573. 10.1128/AAC.04922-14 25779577 PMC4432215

[B36] McGreal E. P. Rosas M. Brown G. D. Zamze S. Wong S. Y. Gordon S.. (2006). The carbohydrate-recognition domain of Dectin-2 is a C-type lectin with specificity for high mannose. Glycobiology 16, 422–430. 10.1093/glycob/cwj077 16423983

[B37] Mora-Montes H. M. Netea M. G. Ferwerda G. Lenardon M. D. Brown G. D. Mistry A. R.. (2011). Recognition and blocking of innate immunity cells by *Candida albicans* chitin. Infect. Immun. 79, 1961–1970. 10.1128/IAI.01282-10 21357722 PMC3088140

[B38] Navarro-Arias M. J. Hernandez-Chavez M. J. Garcia-Carnero L. C. Amezcua-Hernandez D. G. Lozoya-Perez N. E. Estrada-Mata E.. (2019). Differential recognition of *Candida tropicalis, Candida guilliermondii, Candida krusei*, and *Candida auris* by human innate immune cells. Infect. Drug Resist. 12, 783–794. 10.2147/IDR.S197531 31040708 PMC6459152

[B39] Netea M. G. Brown G. D. Kullberg B. J. Gow N. A. (2008). An integrated model of the recognition of *Candida albicans* by the innate immune system. Nat. Rev. 6, 67–78. 10.1038/nrmicro1815 18079743

[B40] Netea M. G. Gow N. A. Munro C. A. Bates S. Collins C. Ferwerda G.. (2006). Immune sensing of *Candida albicans* requires cooperative recognition of mannans and glucans by lectin and Toll-like receptors. J. Clin. Invest. 116, 1642–1650. 10.1172/JCI27114 16710478 PMC1462942

[B41] Pardini G. De Groot P. W. Coste A. T. Karababa M. Klis F. M. de Koster C. G.. (2006). The *CRH* family coding for cell wall glycosylphosphatidylinositol proteins with a predicted transglycosidase domain affects cell wall organization and virulence of *Candida albicans*. J. Biol. Chem. 281, 40399–40411. 10.1074/jbc.M606361200 17074760

[B42] Park S. Kelly R. Kahn J. N. Robles J. Hsu M. J. Register E.. (2005). Specific substitutions in the echinocandin target Fks1p account for reduced susceptibility of rare laboratory and clinical *Candida* sp. isolates. Antimicrob. Agents Chemother. 49, 3264–3273. 10.1128/AAC.49.8.3264-3273.2005 16048935 PMC1196231

[B43] Pfaller M. Neofytos D. Diekema D. Azie N. Meier-Kriesche H. U. Quan S. P.. (2012). Epidemiology and outcomes of candidemia in 3648 patients: data from the Prospective Antifungal Therapy [PATH Alliance(R)] registry, 2004-2008. Diagn. Microbiol. Infect. Dis. 74, 323–331. 10.1016/j.diagmicrobio.2012.10.003 23102556

[B44] Pfaller M. A. Messer S. A. Woosley L. N. Jones R. N. Castanheira M. (2013). Echinocandin and triazole antifungal susceptibility profiles for clinical opportunistic yeast and mold isolates collected from 2010 to 2011: application of new CLSI clinical breakpoints and epidemiological cutoff values for characterization of geographic. J. Clin. Microbiol. 51, 2571–2581. 10.1128/JCM.00308-13 23720791 PMC3719648

[B45] Pham C. D. Iqbal N. Bolden C. B. Kuykendall R. J. Harrison L. H. Farley M. M.. (2014). Role of *FKS* mutations in *Candida glabrata*: MIC values, echinocandin resistance, and multidrug resistance. Antimicrob. Agents Chemother. 58, 4690–4696. 10.1128/AAC.03255-14 24890592 PMC4136002

[B46] Plaine A. Walker L. Da Costa G. Mora-Montes H. M. McKinnon A. Gow N. A.. (2008). Functional analysis of *Candida albicans* GPI-anchored proteins: roles in cell wall integrity and caspofungin sensitivity. Fungal Genet. Biol. 45, 1404–1414. 10.1016/j.fgb.2008.08.003 18765290 PMC2649418

[B47] Porcaro I. Vidal M. Jouvert S. Stahl P. D. Giaimis J. (2003). Mannose receptor contribution to *Candida albicans* phagocytosis by murine E-clone J774 macrophages. J. Leukoc. Biol. 74, 206–215. 10.1189/jlb.1202608 12885937

[B48] Sato K. Yang X. L. Yudate T. Chung J. S. Wu J. Luby-Phelps K.. (2006). Dectin-2 is a pattern recognition receptor for fungi that couples with the Fc receptor gamma chain to induce innate immune responses. J. Biol. Chem. 281, 38854–38866. 10.1074/jbc.M606542200 17050534

[B49] Seider K. Brunke S. Schild L. Jablonowski N. Wilson D. Majer O.. (2011). The facultative intracellular pathogen *Candida glabrata* subverts macrophage cytokine production and phagolysosome maturation. J. Immunol. 187, 3072–3086. 10.4049/jimmunol.1003730 21849684

[B50] Seider K. Gerwien F. Kasper L. Allert S. Brunke S. Jablonowski N.. (2014). Immune evasion, stress resistance, and efficient nutrient acquisition are crucial for intracellular survival of *Candida glabrata* within macrophages. Eukaryot. Cell 13, 170–183. 10.1128/EC.00262-13 24363366 PMC3910963

[B51] Skrzypek M. S. Binkley J. Binkley G. Miyasato S. R. Simison M. Sherlock G. (2016). The *Candida* Genome Database (CGD): incorporation of Assembly 22, systematic identifiers and visualization of high throughput sequencing data. Nucleic Acids Res. 45, D592–D596. 10.1093/nar/gkw924 27738138 PMC5210628

[B52] Stahl P. D. Rodman J. S. Miller M. J. Schlesinger P. H. (1978). Evidence for receptor-mediated binding of glycoproteins, glycoconjugates, and lysosomal glycosidases by alveolar macrophages. Proc. Natl. Acad. Sci. U.S.A. 75, 1399–1403. 10.1073/pnas.75.3.1399 274729 PMC411479

[B53] Sullivan D. J. Westerneng T. J. Haynes K. A. Bennett D. E. Coleman D. C. (1995). *Candida dubliniensis* sp. nov.: phenotypic and molecular characterization of a novel species associated with oral candidosis in HIV-infected individuals. Microbiology 141, 1507–1521. 10.1099/13500872-141-7-1507 7551019

[B54] Taylor P. R. Tsoni S. V. Willment J. A. Dennehy K. M. Rosas M. Findon H.. (2007). Dectin-1 is required for beta-glucan recognition and control of fungal infection. Nat. Immunol. 8, 31–38. 10.1038/ni1408 17159984 PMC1888731

[B55] Thompson A. Griffiths J. S. Walker L. da Fonseca D. M. Lee K. K. Taylor P. R.. (2019). Dependence on Dectin-1 Varies With Multiple *Candida* Species. Front. Microbiol. 10:1800. 10.3389/fmicb.2019.01800 31447813 PMC6691182

[B56] Toth A. Csonka K. Jacobs C. Vagvolgyi C. Nosanchuk J. D. Netea M. G.. (2013). *Candida albicans* and *Candida parapsilosis* induce different T-cell responses in human peripheral blood mononuclear cells. J. Infect. Dis. 208, 690–698. 10.1093/infdis/jit188 23661798 PMC3719900

[B57] Wagener J. Malireddi R. K. Lenardon M. D. Koberle M. Vautier S. MacCallum D. M.. (2014). Fungal chitin dampens inflammation through IL-10 induction mediated by *NOD2* and *TLR9* activation. PLoS Pathog. 10:e1004050. 10.1371/journal.ppat.1004050 24722226 PMC3983064

[B58] Walker C. A. Gomez B. L. Mora-Montes H. M. Mackenzie K. S. Munro C. A. Brown A. J.. (2010). Melanin externalization in *Candida albicans* depends on cell wall chitin structures. Eukaryot. Cell 9, 1329–1342. 10.1128/EC.00051-10 20543065 PMC2937336

[B59] Walker L. A. Gow N. A. Munro C. A. (2013). Elevated chitin content reduces the susceptibility of *Candida* species to caspofungin. Antimicrob. Agents Chemother. 57, 146–154. 10.1128/AAC.01486-12 23089748 PMC3535899

[B60] Walker L. A. Munro C. A. de Bruijn I. Lenardon M. D. McKinnon A. Gow N. A. (2008). Stimulation of chitin synthesis rescues *Candida albicans* from echinocandins. PLoS Pathog. 4:e1000040. 10.1371/journal.ppat.1000040 18389063 PMC2271054

[B61] Wheeler R. T. Fink G. R. (2006). A drug-sensitive genetic network masks fungi from the immune system. PLoS Pathog. 2:e35. 10.1371/journal.ppat.0020035 16652171 PMC1447670

[B62] Wheeler R. T. Kombe D. Agarwala S. D. Fink G. R. (2008). Dynamic, morphotype-specific *Candida albicans* beta-glucan exposure during infection and drug treatment. PLoS Pathog. 4:e1000227. 10.1371/journal.ppat.1000227 19057660 PMC2587227

[B63] Wileman T. E. Lennartz M. R. Stahl P. D. (1986). Identification of the macrophage mannose receptor as a 175-kDa membrane protein. Proc. Natl. Acad. Sci. U.S.A. 83, 2501–2505. 10.1073/pnas.83.8.2501 3458213 PMC323326

