# Supplementary figures and images for: Caspofungin Induced Cell Wall Changes of Candida Species Influences Macrophage Interactions
Source: Front Cell Infect Microbiol. 2020 May 12;10:164. doi: 10.3389/fcimb.2020.00164 (PMC7247809; doi:10.3389/fcimb.2020.00164)

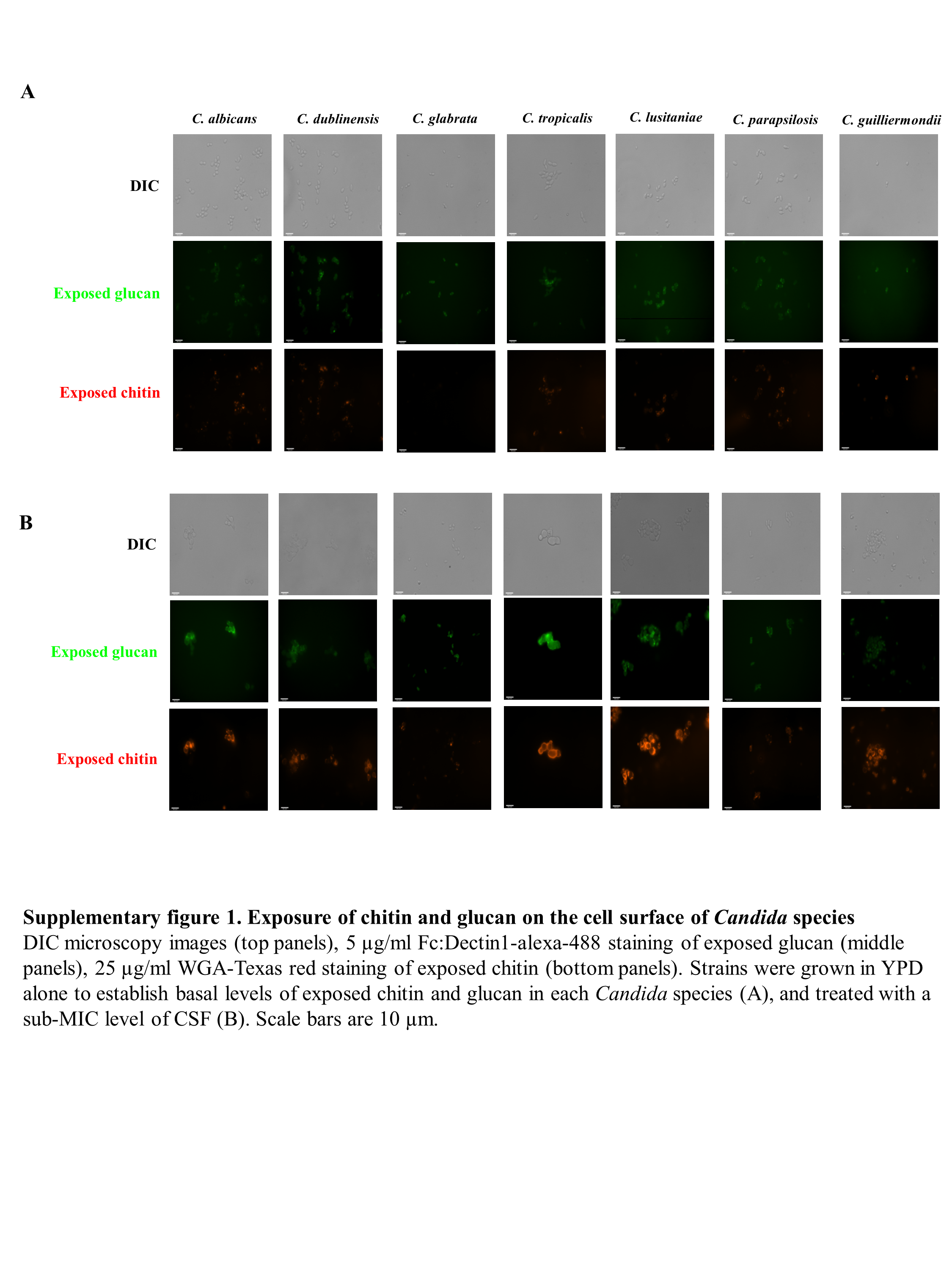

Supplement: Supplementary file 1 [file Image_1.TIF]
